# Shedding Light on Nocturnal Movements in Parkinson’s Disease: Evidence from Wearable Technologies

**DOI:** 10.3390/s20185171

**Published:** 2020-09-10

**Authors:** Alessandro Zampogna, Alessandro Manoni, Francesco Asci, Claudio Liguori, Fernanda Irrera, Antonio Suppa

**Affiliations:** 1 Department of Human Neurosciences, Sapienza University of Rome, 00185 Rome, Italy; alessandro.zampogna@uniroma1.it (A.Z.); francesco.asci@uniroma1.it (F.A.); 2 Department of Information Engineering, Electronics and Telecommunications, Sapienza University of Rome, 00184 Rome, Italy; alessandro.manoni@uniroma1.it (A.M.); fernanda.irrera@uniroma1.it (F.I.); 3 Department of Systems Medicine, University of Rome Tor Vergata, 00133 Rome, Italy; dott.claudioliguori@yahoo.it; 4 IRCCS Neuromed, 86077 Pozzilli (IS), Italy

**Keywords:** parkinson’s disease, nocturnal movements, actigraphy, wearable sensors, polysomnography, akinesia, RBD

## Abstract

In Parkinson’s disease (PD), abnormal movements consisting of hypokinetic and hyperkinetic manifestations commonly lead to nocturnal distress and sleep impairment, which significantly impact quality of life. In PD patients, these nocturnal disturbances can reflect disease-related complications (e.g., nocturnal akinesia), primary sleep disorders (e.g., rapid eye movement behaviour disorder), or both, thus requiring different therapeutic approaches. Wearable technologies based on actigraphy and innovative sensors have been proposed as feasible solutions to identify and monitor the various types of abnormal nocturnal movements in PD. This narrative review addresses the topic of abnormal nocturnal movements in PD and discusses how wearable technologies could help identify and assess these disturbances. We first examine the pathophysiology of abnormal nocturnal movements and the main clinical and instrumental tools for the evaluation of these disturbances in PD. We then report and discuss findings from previous studies assessing nocturnal movements in PD using actigraphy and innovative wearable sensors. Finally, we discuss clinical and technical prospects supporting the use of wearable technologies for the evaluation of nocturnal movements.

## 1. Introduction

Parkinson’s disease (PD) is the fastest-growing neurological disorder in prevalence, disability, and deaths worldwide [1]. PD diagnosis is based on the clinical recognition of specific motor manifestations, including bradykinesia (i.e., movement slowness and amplitude or speed decrement), rigidity (i.e., velocity-independent resistance of major joints to passive movement), and 4–6 Hz rest tremor [2]. Moreover, several non-motor symptoms, including cognitive, sensory, and autonomic dysfunction, invariably contribute to increasing the disease burden on patients and caregivers [3]. Although the loss of dopaminergic neurons in the substantia nigra pars compacta (SNpc) is the main pathophysiological mechanism responsible for the occurrence of most motor symptoms in PD, current consensus attributes several parkinsonian signs and symptoms to neurodegeneration in other neurotransmitter systems, such as cholinergic and noradrenergic pathways. L-Dopa and other drugs directly or indirectly acting on dopaminergic transmission in the central nervous system are the main therapeutic strategies used in PD patients [4,5,6]. 

PD patients commonly experience nocturnal distress and sleep impairment due to the occurrence of several disturbances [7,8]. Among these, abnormal movements consisting of hypokinetic and hyperkinetic manifestations are major contributing factors to nocturnal distress and sleep impairment in PD patients and their bed partners. Abnormal nocturnal movements in PD can reflect disease-related complications, primary sleep disorders, or both [7]. Disease-related complications mostly occur in more advanced PD stages and reflect the complex interaction between chronic L-Dopa intake and the amount of neurodegeneration in the dopaminergic system. Among disease-related nocturnal complications, akinesia is one of the most disabling consequences, leading to abnormal nocturnal movements in PD [9]. Nocturnal akinesia is a hypokinetic disorder consisting of partial or complete loss of movement in the axial body and limbs, limiting body position changes and leading to prolonged immobilization, pain, and sleep impairment [9]. In addition, PD patients can manifest primary sleep disorders, such as restless legs syndrome (RLS), periodic limb movement disorder (PLMD), and rapid eye movement (REM) behaviour disorder (RBD), which, unlike nocturnal akinesia, may occur in early or even prodromal phases of PD [10]. While RLS and PLMD are sleep-related movement disorders primarily characterised by segmental and stereotyped movements, RBD is a more compound syndrome (i.e., parasomnia) associated with complex motor behaviours [11]. Nocturnal akinesia and primary sleep disorders frequently coexist in PD and, due to their specific pathophysiologic mechanisms, require different therapeutic approaches. Accordingly, it is crucial to recognise and classify abnormal nocturnal movements in order to optimise therapeutic strategies in PD patients.

Actigraphy and innovative wearable sensors have increasingly been used to monitor nocturnal movements in PD. Actigraphy is based on the placement of a clock-like instrument, called an actigraph, which contains sensing technologies (e.g., an accelerometer and temperature, noise, and light detectors) able to monitor specific biological functions and reconstruct the sleep/wake cycle [12]. Along with the examination of sleep impairment, some previous studies using actigraphy in PD have also provided information about nocturnal limb movements in parkinsonian patients [13,14,15,16,17,18,19,20,21,22,23,24]. More recently, innovative wearable technologies including wireless sensor networks have also been used to examine nocturnal movements involving axial body rotations in PD patients [25,26,27,28,29,30,31,32,33,34,35,36]. Compared to clinical evaluation and polysomnography, actigraphy and innovative wearable sensors are now increasingly considered feasible solutions for the long-term recording of a large amount of ecological data due to the low costs and unobtrusiveness of these tools.

This narrative review first discusses the issue of abnormal nocturnal movements in PD. We then summarise the main clinical and instrumental tools currently available for the evaluation of nocturnal movements, including polysomnography and wearable technologies. We subsequently report findings from previous studies assessing nocturnal movements in PD patients through actigraphy and innovative wearable sensors. Finally, we discuss the prospects and challenges of wearable technologies in the instrumental assessment of nocturnal movements in PD.

## 2. Pathophysiology of Abnormal Nocturnal Movements in Parkinson’s Disease

Humans spend nearly one-third of their lives sleeping in bed. Optimal nocturnal rest involves the ability to freely move consciously or unconsciously in order to find a comfortable body position. In addition to voluntary movements when awake in bed, dozens of major body shifts and hundreds of small and non-purposeful movements commonly occur in healthy subjects (HS) while sleeping. These movements largely depend on sleep architecture [37]. In healthy adults, sleep architecture reflects the alternation of REM sleep and three progressively deepening stages (i.e., N1, N2, and N3) of non-REM (NREM) sleep. Wakefulness, NREM sleep, and REM sleep show specific physiological characteristics in terms of brain activity, eye movement, and muscle activity, which can be measured by electroencephalography (EEG), electrooculography (EOG), and electromyography (EMG), respectively [38,39] (Table 1).

Concerning physiological nocturnal movements, immobility predominantly characterises descending phases of the sleep cycle (e.g., transition from NREM stage 2 to 3). Conversely, transitions from NREM stage 3 to NREM stage 2 or REM sleep show major position changes [40,41]. Moreover, sleep/wake transitions sometimes present brief and sudden involuntary muscle contractions (i.e., hypnic and propriospinal myoclonus). These movements are considered physiological phenomena when they do not affect sleep through arousal and fragmentation [42]. REM sleep is usually characterised by shorter and jerkier movements as compared to NREM sleep [43]. Small movements mainly involve the lower extremities and are more frequent in men than women [43]. Sleep-related motor activity before sleep onset or during sleep can also include rhythmic and stereotyped movements, such as head rocking or body rolling, though these movements are much more common in infants than adults [44]. Ageing is an important factor impacting sleep-related motor activity in HS. Indeed, nocturnal movements commonly decrease over time, likely reflecting decreased daytime motor activity in the elderly [45]. Accordingly, young adults have more segmental body movements and greater position changes than older adults during sleep [45,46,47]. Furthermore, elderly adults have increased movement distribution across sleep stages and reduced motor activity during REM sleep as compared to young adults [46,47].

In addition to age-related sleep changes, pathophysiologic findings of abnormal nocturnal movements in PD patients include abnormal sleep macrostructure (e.g., sleep fragmentation and sleep-cycle dysregulation with a relative increase in superficial sleep), and impaired sleep microstructure (e.g., disturbed sleep spindles and K-complexes) [48]. Accordingly, sleep architecture changes may partially contribute to abnormal nocturnal movements in PD. Moreover, abnormal nocturnal movements in PD may crucially reflect neurodegenerative progression in the central nervous system. When cardinal motor signs appear in PD patients, up to 60–70% of dopaminergic neurons in the SNpc have already been lost [49,50]. The surviving dopaminergic neurons can still store and continuously release dopamine in the synaptic cleft, thus allowing a long-duration response to the exogenous administration of L-Dopa (honeymoon period) [51]. However, as the disease progresses, dopaminergic neuronal loss worsens endogenous dopamine synthesis, storage, and release, leading to a progressive decline in the long-duration response to exogenous administration of L-Dopa [51]. As a result, PD patients in more advanced stages of the disease require additional daily administrations of L-Dopa to achieve stable improvement of motor symptoms due to the short-duration response to exogenous L-Dopa, which directly depends on L-Dopa plasmatic levels [51]. Accordingly, when partial nighttime dopaminergic drug withdrawal leads to akinesia, PD patients may progressively experience nocturnal distress and sleep impairment.

The second main cause of abnormal nocturnal movements in PD is primary sleep disorders, including RBD, RLS, and PLMD, which reflect dysfunction of sleep regulatory centres in the pons and medulla, implicating noradrenergic and cholinergic dysfunction [52,53,54]. According to the well-known progression of neurodegenerative processes in PD [55], RBD frequently precedes cardinal parkinsonian motor symptoms [56]. Table 2 summarises the main causes of abnormal nocturnal movements and reports the main clinical manifestations and pathophysiological mechanisms in PD.

## 3. Clinical Assessment of Nocturnal Movements

Clinical assessment in PD patients commonly involves individual interviews, diaries, and rating scales to investigate nocturnal disturbances, including abnormal movements. All these approaches are patient-based instruments and thus examine subjective perceptions of symptoms with variable psychometric properties. Rating scales are the only standardised clinical tools for the semiquantitative evaluation of nocturnal symptom severity and impact on quality of life. These scales investigate a wide range of sleep disturbances, including insomnia, nocturia, breathing disorders, and daytime sleepiness [59]. However, the most relevant scales commonly used in PD only partially address the issue of abnormal nocturnal movements (Table 3) [59].

When considering the clinical assessment of nocturnal movements in PD, several limitations should be considered. First, PD patients are often unaware of specific nocturnal disorders affecting sleep, such as apnoea, PLMD, and RBD. Second, poor recall memory can also affect the reliability of data collection. Third, cognitive impairment and other neuropsychiatric disorders may preclude the administration of standardised clinical scales. Fourth, all these limitations also affect information reported by caregivers and/or bed partners. Overall, these drawbacks in the clinical evaluation of abnormal nocturnal movements in PD underline the need for instrumental examination of nocturnal disturbances.

## 4. Instrumental Assessment of Nocturnal Movements

### 4.1. Polysomnography

Polysomnography is the current gold standard for the assessment and diagnosis of sleep disturbances. Polysomnography includes specific instrumentation for the evaluation of nocturnal movements [60] and is based on standardised methods requiring hospital-based settings, compound instrumentation, and trained technicians. Polysomnography contains multiple tools, including EEG, EOG, oronasal thermal sensors, respiratory inductance plethysmography, pulse oximetry, and single-lead electrocardiography. To assess nocturnal movements, polysomnography usually uses video recordings and a body-position sensor to provide information on overnight body position changes. Moreover, surface EMG (sEMG) of the mental muscle and flexor or extensor digitorum muscles in the forearms is recorded to assess muscle tone during REM sleep. Finally, sEMG of the anterior tibialis muscles is also used to monitor limb movements and possibly identify a periodic pattern. By monitoring several physiological functions, polysomnography allows sleep structure to be studied and different stages of NREM and REM sleep to be identified, thus providing a graphic representation, termed the “hypnogram”. Polysomnography also provides several quantitative parameters to assess sleep features, such as sleep latency (i.e., sleep onset delay), wake after sleep onset, sleep efficiency (i.e., total sleep time divided by total recording time), sleep stage percentages, periodic limb movement index, arousal index, and cyclic alternating pattern (i.e., a marker of sleep stability and continuity) [60].

However, polysomnography has some limitations that restrict its use. It is a cumbersome, labour-intensive, and expensive examination due to the high resource burden required for data acquisition. Moreover, since the laboratory setting and technical equipment do not reflect regular sleep conditions in a domestic environment, polysomnography provides poor ecological data. Indeed, specific external factors commonly occur at home and possibly affect patient sleep. Furthermore, a one-night assessment with polysomnography may be insufficient for the patient to become comfortable with the examination setting (i.e., first-night effect), and a time-limited recording could miss sporadic pathological events. Although polysomnography is also available for domestic assessment, this method requires high patient collaboration due to composite equipment and may provide suboptimal accuracy as compared to in-laboratory measurements [15,16]. The huge demand for polysomnography supports the use of alternative approaches able to provide unobtrusive, ecological, and long-term evaluations [61].

### 4.2. Actigraphy and Innovative Wearable Sensors

Wearable technologies consist of smart electronic devices worn on the body that record different physical and physiological signals, such as heart rate, muscle activity, and body motion. Among wearable technologies, actigraphy and innovative wireless sensors, including inertial and electromyographic devices, are the main tools used to investigate nocturnal movements.

Actigraphy is a simple non-invasive examination that measures body movements multiple times per second, 24 h a day. The actigraph is usually worn on the non-dominant wrist and includes an accelerometer to record linear accelerations. The accelerometer outputs an analogue electrical voltage in response to the motion. This signal is sampled with a specific sampling frequency, usually 10 Hz, and processed in three different ways in order to gather information about movement frequency, duration, and intensity. All extracted information is then saved as data points, with a one-minute interval between each sample and the following one [12]. As a result, about 1440 wrist-movement data points are recorded every 24 h [62]. The most widely used method for sleep recognition based on motor activity level is the Zero Crossing Mode, which measures how many times per minute the voltage signal crosses a specific threshold value (usually close to zero) [62]. Moreover, most actigraphic devices have a dedicated button to allow the patient to mark time in and out of bed, making it easier and more accurate to identify the time in bed. There are no contraindications to performing actigraphy, and its execution does not require any specific preparation or setup. Therefore, it is possible to examine the sleep/wake cycle, circadian rhythms, and sleep- and wake-related disorders through actigraphy. In addition, by integrating different signals and additional sensing components, such as temperature, noise, and light detectors, actigraphy can provide more information about the quality of the sleep environment. Data collected through actigraphy can be downloaded on a personal computer and processed using a dedicated algorithm that separately assesses sleep/wake cycles and sleep parameters. Like polysomnography, the algorithm provides a wide variety of parameters and sleep indices, such as total sleep time, sleep efficiency (i.e., the percentage of time spent sleeping when in bed), sleep latency, wake after sleep onset, fragmentation index (i.e., the number of sleep interruptions), and number of awakenings [63]. Furthermore, the elaboration of actigraphic data offers several movement measures, including mean and median activity during time in bed (i.e., nocturnal activity reflected by the number of wrist movements), movement index (i.e., the number of limb motions divided by time in bed), mean duration of immobility periods, ratio of nighttime and daytime activity, and motility time. Overall, the negligible obtrusiveness and high simplicity of actigraphy allow prolonged monitoring without a significant impact on patient behaviour, thus offering valuable ecological data. However, despite its extensive application in sleep research, experimental evidence specifically addressing the methodology of sleep parameter estimation through actigraphy is currently limited.

Innovative wearable sensors include advanced electronic devices that share several features with actigraphy, such as small size, low-to-no manual intervention, and very low costs. Besides actigraphy, one of the most widely used embedded sensors to analyse movement is the triaxial accelerometer. By measuring linear acceleration on three different reference axes, the accelerometer allows an accurate reconstruction of patient movements and positions. In addition to an accelerometer, these devices also commonly include a gyroscope measuring angular velocity and sometimes a magnetometer to analyse magnetic field changes. Inertial measurement units (IMUs) are electronic sensors that use a combination of accelerometers and gyroscopes to provide three-dimensional information on body motion and position.

Unlike actigraphy, innovative wearable sensors offer versatile solutions regarding placement. Patients can also wear multiple devices, thus creating a body area network (BAN). In a BAN, sensors are typically time-synchronised and regulated by a master control unit that reorders data from multiple slave devices before transmission to a computer. Both intra-BAN and inter-BAN communication protocols are critical to provide a reliable output, though these protocols become more complex and challenging as the number of sensors in the BAN increases [64]. With BANs, it is possible to have a partial or complete human avatar that provides a visual reconstruction of body movements and positions [65].

**Figure 1:**
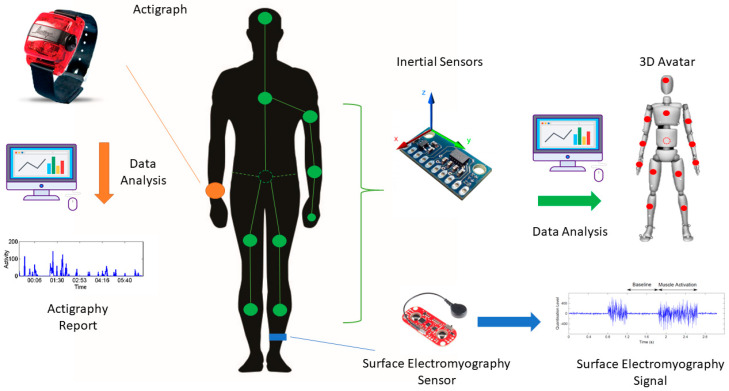
Wearable technologies for the assessment of nocturnal movements. On the left, a representative actigraph placed on the wrist (orange dot) able to record linear accelerations for data analysis through dedicated algorithms. On the right, innovative wearable devices consisting of inertial and surface electromyography sensors. Multiple inertial sensors can be used to create a body area network (green dots and lines) providing three-dimensional information on body motion and position (e.g., 3D Avatar). Surface electromyography sensors record muscle activity that can be integrated with kinematic data through a “sensor fusion” approach.

A more recent wearable technology approach involves the integration of different sensory data, such as inertial and EMG signals. This approach, also termed “sensor fusion”, provides more complete information on body motion, including data on muscle activity through sEMG wireless sensors [66]. Accordingly, innovative devices composed of both IMUs and sEMG are increasingly used to optimize the objective recognition of motor disorders in PD patients [67,68]. When using innovative wearable sensors, it is essential to consider also some limitations. Concerning IMUs, possible technical challenges include magnetic interferences, energy consumption, calibration loss, and errors of misalignment, orthogonality, and offset. Moreover, skin-electrode interface noise and crosstalk due to adjacent muscles may also affect recordings through sEMG sensors [68]. Figure 1 reports the main wearable technologies available for nocturnal movement assessment, including the actigraph and inertial and sEMG sensors.

## 5. Literature Search Strategies and Criteria

Three independent researchers (A.Z., A.M., and F.A.) performed a literature search of studies investigating nocturnal movements in PD through wearable technologies using Medline, Scopus, PubMed, Web of Science, EMBASE, and Cochrane Library databases. All possible combinations of the following keywords, including hyphens and inverted commas, were used: “Parkinson’s disease” OR “parkinsonian” OR “parkinsonism” AND “sensors” OR “wearable technologies” OR “accelerometer” OR “gyroscope” OR “IMU” OR “wireless device” OR “actigraphy” OR “sEMG” AND “nocturnal movements” OR “hypokinesia” OR “akinesia” OR “sleep” OR “turning in bed” OR “hyperkinesia” OR “primary sleep disorders” OR “sleep-related movements” OR “RBD” OR “RLS” OR “PLMD”. Experimental studies on wearable technology recordings published before July 2020 were considered for eligibility. The references of each matched article were also examined in order to include relevant studies that were not identified in the electronic databases. We excluded reviews, reports, and articles in languages other than English from our literature search. We first collected eligible studies based on title and abstract. We then evaluated full texts according to the inclusion and exclusion criteria. Finally, we extracted the demographic and clinical features of subjects enrolled in the selected studies, as well as information on sensor type and location, use of polysomnography, monitoring duration, and main sensor-based outcome measures, including clinical-behavioural correlations.

## 6. Nocturnal Movements in Parkinson’s Disease: Studies Using Wearable Technologies

### 6.1. Actigraphy

By monitoring the sleep/wake cycle, actigraphy has become the most widely used wearable technology for the assessment of nocturnal disturbances in PD patients. Although this approach relies on the measurement of limb motor activity, only a limited number of studies have specifically assessed nocturnal movements in PD through actigraphy [13,14,15,16,17,18,19,20,21,22,23,24]. Conversely, most authors used this tool to indirectly examine sleep quality through specific measures, such as the fragmentation index and sleep latency, time, and efficiency. Overall, studies that used actigraphy in PD addressed the following four main issues: (i) assessment of sleep features [13,16,17,20,21,22,23,24,69,70,71,72,73,74,75,76,77,78,79,80,81,82,83,84,85,86,87,88]; (ii) evaluation of therapeutic interventions [14,15,83,89,90,91,92,93,94]; (iii) validation of clinical scales [95,96], and; (iv) identification of specific primary sleep disorders [18,19].

Concerning device placement, most authors placed the actigraph on the upper limbs of PD patients, predominantly on the least affected side in order to avoid possible recording bias due to motor symptoms such as tremor. However, several authors did not consider this issue and used a priori the non-dominant wrist or lower limb. One study compared actigraphic recordings between the upper and lower limbs and found differences in several parameters (e.g., sleep efficacy and total sleep time) [82]. The unobtrusiveness of actigraphy has allowed long-duration evaluations of the sleep/wake cycle for up to 28 days in PD patients.

Regarding actigraphic measures, the most widely used parameters include total nocturnal moving time, derived indices such as the movement index, and static body position distribution. Several authors have found increased nocturnal motor activity during rest in PD patients as compared to HS, especially in those with hallucinations [13,20,21,23,24]. Conversely, one study demonstrated lower motor activity in early PD patients than in HS [16]. Despite a similar frequency of body turns when awake in bed, PD patients complaining of impaired bed mobility had reduced body position changes during sleep as compared with PD patients who did not report this symptom [17]. A few authors assessed primary sleep disorders associated with abnormal nocturnal movements in PD, such as RBD and RLS [18,19,22]. These studies demonstrated a low sensitivity of actigraphy in the identification and classification of primary sleep disorders. Unexpectedly, actigraphy recorded a comparable number of nocturnal movements in PD patients with and without RBD [18]. Actigraphy identified the two patient subgroups with good specificity but very low sensitivity by comparing the total number of wake bouts, which was higher in patients with RBD [18,19]. Moreover, lower limb actigraphy failed to disclose a significant association between periodic limb movements and RLS in PD [22]. Finally, two studies assessing the impact of pergolide and deep brain stimulation on nocturnal movements in PD [14,15] demonstrated that the former increased the movement index during sleep [15], whereas the latter did not [14].

Previous studies assessing possible clinical-behavioural correlations found no associations between the periodic limb movement index and RLS in PD patients. In contrast, some actigraphic measures, such as total rest time and number of wake bouts, positively correlated with the REM Sleep Behaviour Disorder Screening Questionnaire.

Considering its accuracy in sleep assessment, a limited number of authors have also used actigraphy in combination with polysomnography in PD [14,17,18,20,72,73,75,76,79,89,94] and found a fairly high agreement rate on specific measures, such as sleep time and efficiency. However, agreement between the two techniques has not been evaluated with respect to nocturnal movements in PD [20,73,79]. Several factors, including disease stage, increase sleep recording variability, thus worsening actigraphic performance [73]. Table 4 summarises studies that have used actigraphy for sleep assessment in PD, including those examining nocturnal movements.

### 6.2. Innovative Wearable Sensors

A small number of authors have used innovative wearable sensors to investigate abnormal nocturnal movements in PD [25,26,27,28,29,30,31,32,33,34,35,97]. A single research group has performed most of these investigations (7 out of 12 studies) [26,27,28,29,30,31,34]. Overall, these studies have examined a heterogeneous cohort of PD patients, including subjects with early and advanced disease stages. Only 8 out of 12 studies also enrolled HS [25,26,27,29,31,34,35,98]. Of the studies that did not enrol HS [28,30,32,33], one investigated changes in nocturnal movements based on disease stage and severity [32], while another investigated subjective perception of impaired bed mobility [33]. Two other studies assessed the effect of different therapeutic approaches (i.e., subcutaneous apomorphine infusion and the rotigotine transdermal patch) to improve nocturnal hypokinesia in PD patients [28,30].

Concerning the type and placement of wearable sensors, all authors adopted inertial devices, including accelerometers and gyroscopes. Most studies used a single inertial sensor placed on the trunk [27,28,29,30], waist [25,35,98], or abdomen [32]. Conversely, a few authors monitored nocturnal movements through a wireless sensor network composed of five inertial devices located on the wrists, ankles, trunk, or abdomen [26,31,33,34]. Overnight sensor-based recordings lasted a limited time that was restricted to one or two nights.

Regarding output measures, all authors examined body rotations by analysing the number, velocity, acceleration, and degree of axial turns in bed [25,26,27,28,29,30,31,32,33,34,35,98]. Moreover, some studies also assessed overnight body positions [27,31,35], number of limb movements [26,31,33], and frequency of getting out of bed [26,27,28,29,31,33]. Concerning body rotations, while several studies found a reduced number of nocturnal turns in PD patients compared to HS [26,27,29,31], a few authors demonstrated a similar frequency in the two groups [25,35]. Conversely, all studies consistently found reduced nocturnal body rotation velocity, acceleration, and amplitude in PD patients compared with HS [25,26,27,29,31,35,98]. Regarding nocturnal body positions, a few studies demonstrated that PD patients usually spent more time in the supine position [31,35] and less frequently manifested postural changes compared to HS [27]. Moreover, only one study compared the number of limb movements in PD patients and HS, demonstrating a similar amount of activity in the two groups [31]. Lastly, some studies reported an increased [26,27] or similar [31] frequency of getting out of bed in PD patients compared to HS. Overall, nocturnal movement changes were prominent in PD patients with advanced disease stages [32,35] and in those with subjective perception of impaired bed mobility [33], especially during the second half of the night [31,35]. Subcutaneous apomorphine infusion and the rotigotine transdermal patch partially increased body axial rotation in PD patients [28,30].

Several authors have demonstrated correlations between specific clinical features, such as disease duration, severity, and axial impairment; and behavioural measures, such as body rotations (e.g., degree, velocity, and acceleration) [26,27,28,29,31,32,33,34,35]. Moreover, one study also demonstrated that nocturnal bed rotations in PD patients not only correlated with motor disturbances, but also with cognitive function and non-motor symptoms [35].

No authors measured the accuracy of innovative wearable sensors in the investigation of abnormal nocturnal movements in PD. One previous study combined innovative wearable sensors with polysomnography, but did not compare the performance of the two systems in monitoring nocturnal movements in PD [33]. Table 5 summarises the main data of studies that have used innovative wearable sensors to assess nocturnal movements in PD.

## 7. Discussion

**Figure 2:**
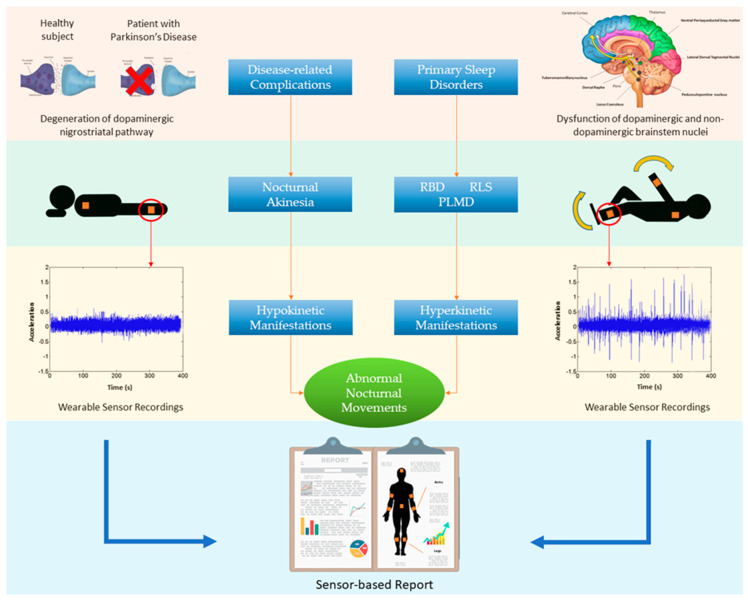
Abnormal nocturnal movements in Parkinson’s Disease (PD). Disease-related complications due to degeneration of the dopaminergic nigrostriatal pathway lead to nocturnal akinesia that is responsible for hypokinetic manifestations (left side). Primary sleep disorders, including rapid eye movement behaviour disorder (RBD), restless legs syndrome (RLS), and periodic limb movement disorder (PLMD), are associated with dysfunction of dopaminergic and non-dopaminergic brainstem nuclei, and lead to hyperkinetic manifestations (right side). Hypo and hyperkinetic manifestations frequently coexist in PD and can be recorded through wearable sensors that provide information about frequency, duration, severity, and body distribution of abnormal nocturnal movements.

In the present review, we addressed the topic of abnormal nocturnal movements in PD by examining previous studies using actigraphy and innovative wearable sensors. Overall, these studies have shown several abnormal nocturnal movements in PD patients, including both hypo and hyperkinetic manifestations (Figure 2).

Our discussion of specific clinical issues and current instrumental tools provides an up-to-date and detailed review of studies using advanced technologies in order to foster a better understanding of abnormal nocturnal movements in PD.

### 7.1. Actigraphy

Since the main scope of actigraphy is to examine rest-activity cycles based on the amount of motor activity rather than nocturnal movements, only limited and often indirectly deduced data are currently available in PD.

A first consideration concerns actigraph placement in PD patients. Only one study has compared recordings from different body regions of PD patients. This study found different measures in the upper and lower limbs but similar results between the two body sides [82]. This finding appears to contrast with the typically asymmetric symptoms in PD patients, possibly because the study did not compare the more and less-affected sides [82]. Hence, the use of a single actigraph may under or overestimate nocturnal movements in PD patients. Accordingly, multiple devices should be placed in order to properly assess nocturnal movements in PD patients.

Most studies have reported higher nocturnal activity in PD patients than HS [13,20,21,23,24], a finding apparently in contrast to the well-known reduced bed mobility in PD secondary to nocturnal akinesia [9]. This inconsistency may reflect the inability of actigraphy to accurately distinguish between abnormal nocturnal movements and voluntary motor activity during nighttime wakefulness. Moreover, actigraphy only partially discriminates isolated limb movements from axial turns or position changes involving multiple body segments. Hence, actigraphy may not properly classify abnormal nocturnal movements in PD [18,19].

To date, although a few studies have compared actigraphic performance with polysomnography recordings in the evaluation of specific sleep parameters in PD [20,73,79], none have assessed the accuracy of actigraphy in evaluating nocturnal movements.

Overall, although actigraphy is objective, ecological, and unobtrusive, it provides limited information regarding nocturnal movements in PD. Future studies are necessary to provide specific recommendations for actigraph placement and to evaluate the accuracy of this method in assessing nocturnal movements in PD.

### 7.2. Innovative Wearable Sensors

Based on the motor behaviour to be measured, researchers have applied different numbers of wearable sensors rather than adopting a single standardised placement, which is usually required for actigraphy. All authors analysed patient rotations in bed using at least one inertial sensor placed on the body axis, i.e., the trunk, waist, and/or abdomen [25,26,27,28,29,30,31,32,33,34,35,98]. Authors that also addressed limb movements in PD placed wearable sensors on the limbs, thus possibly involving a BAN [26,31,33]. Unlike actigraphy, the most recent and innovative wearable sensors allow specific motor behaviours to be identified during nighttime in PD. To date, no authors have used sEMG sensors to evaluate nocturnal motor activity in PD. However, monitoring muscle activity through sEMG sensors in addition to inertial signals could significantly contribute to the characterisation of nocturnal motor activity in PD. For instance, since REM sleep usually shows muscle atonia, sEMG sensors could provide additional information about recorded sleep types based on muscle signals.

Regarding findings from studies using innovative wearable sensors, the most relevant results concern the reduced size, velocity, and acceleration of body rotations in bed in PD patients, even in early stages of the disease, as compared to HS [25,26,27,29,31,35,98]. Moreover, some authors have also reported a reduced frequency of these movements [26,27,29,31]. Overall, these findings are consistent with the high prevalence of nocturnal akinesia in PD, which prominently involves the body axis through the worsening of trunk rigidity and bradykinesia. These nocturnal changes were prominent in the second half of the night [31,35] and in patients with a more advanced disease stage [32,35], consistent with the pathophysiology of nocturnal akinesia. In further support of these findings, overnight dopaminergic stimulation through subcutaneous apomorphine infusion or the rotigotine transdermal patch led to an improvement in nocturnal axial turns in PD [28,30]. It is reasonable to assume that patients spend more time in the supine position due to nocturnal akinesia [31,35]. The supine position worsens sleep-related breathing disorders, such as obstructive sleep apnoea syndrome [99]. Accordingly, new therapeutic strategies that improve nocturnal akinesia could also improve some non-motor disorders in PD.

An important current limitation concerns the lack of sensor-based studies on primary sleep disorders, such as RBD, RLS, and PLMD. Only one study has compared limb movements in PD patients and HS, unexpectedly reporting a similar amount of movements in the two groups [31]. This finding appears to contrast with the increased prevalence of primary sleep disorders associated with hyperkinetic manifestations in PD. However, the authors excluded PD patients with RLS and PLMD, which may explain this inconsistency [31].

As for actigraphy, the most advanced wearable sensors currently lack strong validation supporting their use in the assessment of nocturnal movements in PD. Indeed, only one study has combined polysomnography and sensor-based recordings, though this study did not compare the performance of the two systems. Accordingly, despite the availability of multiple sensor-based measures, it is currently difficult to select and compare derived indices for optimal evaluation of nocturnal movements in PD. Furthermore, another relevant issue concerns the lack of standardised reference values reflecting the severity of abnormal nocturnal movements recorded through innovative wearable sensors.

Overall, current evidence supports the use of the most recent wearable sensors to quantitatively measure nocturnal hypokinesia in PD patients at home. However, several issues, including the validation and assessment of primary sleep disorders, remain unresolved and should thus be addressed by future studies. Similarly, the accuracy of advanced wearable sensors in measuring nocturnal movements in PD should also be clarified.

## 8. Conclusions and Prospects

Wearable technologies, including actigraphy and innovative sensors, are useful tools to evaluate nocturnal movements in PD patients and may provide alternative solutions to conventional polysomnography. To date, studies using wearable technologies have allowed instrumental and extensive investigation of abnormal nocturnal movements secondary to akinesia in PD patients, but not of those related to primary sleep disorders. Among wearable technologies, innovative sensors are likely more reliable than actigraphy in recognising and classifying nocturnal movements in PD patients. Based on current evidence, an interconnected network of wearable inertial sensors may be the most suitable solutions to quantify nocturnal akinesia in PD by three-dimensionally reconstructing body motion and position. However, innovative wearable sensors are limited by their inability to identify different sleep stages and specific primary sleep disorders, such as RBD. To overcome these limitations, new advanced approaches based on multimodal sensor systems (e.g., tattoo-type EEG electrodes, sEMG, and photoplethysmography) may allow the proper reconstruction of sleep architecture [100,101,102,103]. Furthermore, multimodal sensor systems and dedicated machine learning algorithms may make it possible to automatically classify sleep stages and recognise abnormal nocturnal movements and primary sleep disorders in PD. These advanced technologies could also make it possible to monitor large amounts of data in an ecological setting over a long period. Lastly, the use of miniaturized and flexible biosensors able to measure L-Dopa or dopamine concentrations through microneedle skin patches could aid in the investigation into the pathophysiology of abnormal nocturnal movements in PD patients [104].

## Figures and Tables

**Table 1:** Physiological features of sleep in older adults.

	Wakefulness	NREM Sleep (75–85% TST)			REM Sleep (15–25% TST)
		N1 (5–10%)	N2 (45–55%)	N3 (15–20%)	
**Brain activity (EEG)**	High (predominant beta rhythm)	Slowing down (rhythmic alpha and theta waves)	Mostly slow (sleep spindles and K complex)	Slow (high-voltage delta waves)	High (desynchronized, low-voltage, mixed-frequency waves and sawtooth waves); dreaming
**Eye movements (EOG)**	Voluntary gaze movements	Slow eye rolling	Rare	Rare	Rapid eye movement
**Muscle activity (EMG)**	High muscle activity	Moderate - low	Low -moderate	Low	Muscle atonia, except for short episodes of phasic muscle activation and limb twitches
**Autonomic activity**	Variable according to environmental changes	Reduced	Reduced	Reduced	Increased

EEG: electroencephalography; EMG: electromyography; EOG: electrooculography; N1: stage 1; N2: stage 2; N3: stage 3; TST: total sleep time.

**Table 2:** Abnormal nocturnal movements in Parkinson’s Disease.

	Disease-Related Complications	Primary Sleep Disorders		
**Nocturnal disturbance**	Akinesia [9]	RBD [52]	RLS [57]	PLMD [58]
**Definition**	Difficulty moving in bed during nighttime	REM parasomnia is responsible for prominent motor activity. Patients physically act out their dreams, often injuring themselves and waking up	A sensorimotor disorder responsible for the overwhelming urge to move when resting, possibly delaying sleep onset	A sleep-related movement disorder responsible for involuntary movements disrupting sleep, mainly during NREM sleep
**Movements**	Partial or complete loss of movement in axial body and limbs	Loss of atonia; complex motor behaviours (e.g., punching, kicking, and screaming)	Voluntary movements, predominantly involving the legs, providing temporary relief and frequently associated with periodic leg movements	Repetitive and rhythmic movements (at least four), lasting 0.5–10 s (e.g., rhythmic extension of the big toe, dorsiflexion of the foot, and flexion of the knee and hip)
**Pathophysiology**	Striatal dopamine depletion	Neurodegeneration in brainstem nuclei (e.g., locus coeruleus, peduncle-pontine nucleus)	Abnormal dopaminergic neurotransmission, hypocretin levels, and brain iron metabolism	Dynamic oscillating changes in spinal and supraspinal structures

NREM: non-rapid eye movement; PLMD: periodic limb movement disorder; RBD: rem behaviour disorder; REM: rapid eye movement; RLS: restless legs syndrome.

**Table 3:** Rating scales evaluating nocturnal movements in Parkinson’s Disease.

Clinical Rating Scales	Description	Items evaluating Nocturnal Movements
IRLS	10 items for the assessment of RLS symptoms and impact on quality of life	All items concern RLS
MDS-UPDRS	Four parts comprising 50 questions for a comprehensive assessment of non-motor and motor symptoms	Item 2.9 (turning in bed); Item 2.11 (getting out of bed)
NMSQ	30 questions for the assessment of non-motor symptoms	Item 9 (getting out of bed for urination); Item 25 (acting out dreams); Item 26 (urgency to move when resting)
NMSS	30 items for the assessment of non-motor symptom severity and frequency	Item 2.6 (urgency to move when resting)
PDSS	15 items for the assessment of sleep disturbances	Item 4 (leg or arm restlessness when resting); Item 5 (fidgeting in bed); Item 8 (getting out of bed for urination); Item 12 (painful posturing of arms or legs)
PDSS-2	15 questions on the frequency and severity of nocturnal disturbances	Item 4 (leg or arm restlessness when resting); Item 5 (urgency to move when resting); Item 8 (getting out of bed for urination); Item 9 (nocturnal hypokinesia); Item 12 (painful posturing of the arms or legs)
PSQI	24 total questions for the assessment of overall sleep quality (5 questions rated by the bed partner or roommate and not included in the final score)	Item 5c (getting out of bed for urination); Item 10c (leg twitching or jerking rated by the bed partner)
RBDSQ	10 questions for the assessment of RBD	All items concern RBD
RBD1Q	Single-question screen for RBD	Single item (acting out dreams)
SCOPA-Sleep	11 items for the assessment of nighttime sleep (5 items) and daytime sleepiness (6 items)	No items

IRLS: International Restless Legs Scale; MDS-UPDRS: Movement Disorder Society-sponsored revision of the Unified Parkinson’s Disease Rating Scale; NMSQ: Non-Motor Symptoms Questionnaire; NMSS: Non-Motor Symptoms Scale; PD: Parkinson’s disease; PDSS: Parkinson’s Disease Sleep Scale; PSQI: Pittsburgh Sleep Quality Index; RBD: REM behaviour disorder; RBDSQ: REM Behaviour Disorder Screening Questionnaire; RBD1Q: REM Sleep Behaviour Disorder Single-Question Screen; SCOPA-Sleep: Scales for Outcomes in Parkinson’s Disease-Sleep Disturbances.

**Table 4:** Actigraphy in Parkinson’s Disease.

[Ref]	Study Aim	PSG	Actigraphy Location	Monitoring Duration	Outcome Measures	Main Findings	Clinical-Behavioural Correlations
[13,16,17,20,21,22,23,24,69,70,71,72,73,74,75,76,77,78,79,80,81,82,83,84,85,86,87,88]	Assessment of sleep features	[17,20,72,73,75,76,79]	Non-dominant wrist, least or most affected arm, lower limbs	1 to 14 days	Nocturnal activity; movement and fragmentation indices; mean duration of immobility periods; ratio of nighttime and daytime activity (relative amplitude); sleep latency, time, efficiency; WASO; daytime napping	Linear correlation between actigraphy and PSG measurements partially dependant on disease stage; higher sleep efficiency and total sleep time when recording lower limbs compared to upper limbs. Higher nighttime activity level, daytime napping, and movement and fragmentation indices; worse WASO and sleep efficiency, latency, and time in PD than HS. Worse sleep measures in patients with advanced PD, mild cognitive impairment, hallucinations, and probable RBD	Actigraphic sleep measures correlated with disease stage and severity, PDSS sleep quality, non-motor symptoms, morning mobility, LEDD, cognitive function, and melatonin blood concentration. No significant associations between the periodic limb movement index and restless legs syndrome
[14,15,83,89,90,91,92,93,94]	Evaluation of therapeutic intervention	[14,89,94]	Non-dominant wrist or least affected arm	14 to 28 days	Total time in bed; movement and fragmentation indices; nocturnal immobility onset and offset times; and sleep latency, time, and efficiency	Good agreement rate (0.85) between actigraphy and PSG for sleep time. LCIG infusion, DBS, melatonin, parietal rTMS and bright light therapy improved actigraphic measures (e.g., sleep quality, latency, duration, fragmentation, and efficiency) during sleep in PD. Dopaminergic therapy was associated with earlier waking and immobility offset times. Pergolide worsened sleep efficiency and fragmentation	Nighttime psychotic symptoms and daytime somnolence correlated with awakening times
[95,96]	Validation of clinical scales	NA	Non-dominant wrist	7 days	Total time in bed; total sleep time; time spent in the awake state; nocturnal activity and motility time	NMSQ, sleep logs, and PDSS appropriately detect sleep disturbances	NMSQ sleep-fatigue questions, sleep logs, and PDSS correlated with actigraphic measures
[18,19]	Identification of sleep disorders	[18]	Least affected arm	7 to 14 days	Number of wake bouts, duration of awakenings	A higher number of wake bouts in PD with RBD than in PD without RBD; actigraphy has high specificity but low sensitivity in the diagnosis of RBD compared to PSG	Total rest time and number of wake bouts positively correlated with RBDSQ

DBS: deep brain stimulation; LCIG: L-Dopa/carbidopa intestinal gel; LEDD: L-Dopa equivalent daily dose; NA: not available; NMSQ: Non-Motor Symptoms Questionnaire; PD: Parkinson’s disease patients; PDSS: Parkinson’s Disease Sleep Scale; PSG: polysomnography; RBD: rapid eye movement sleep behaviour disorder; RBDSQ: REM Sleep Behaviour Disorder Screening Questionnaire; rTMS: repetitive transcranial magnetic stimulation; UPDRS: Unified Parkinson’s Disease Rating Scale; WASO: wake after sleep onset.

**Table 5:** Innovative wearable sensors in Parkinson’s Disease.

Study	Subjects (Mean Age ± SD)	H&Y (Mean ± SD)	PSG	Type and Location of Sensors	Monitoring Duration	Outcome Measures	Main Findings	Clinical-Behavioural Correlations
Yoneyama et al., 2013 [97]	1 PD (60) 2 HS (60 ± 5.7)	3 (OFF state) 2 (ON state)	Not performed	1 triaxial accelerometer on the waist	One night	Turnover angle (body axial rotations)	Smaller turnover angle in PD than HS	Not performed
Louter et al., 2015 [25]	11 PD (65 ± NA) 13 HS (64 ± NA)	1.9 ± 0.3	Not performed	1 inertial device (3 orthogonal accelerometers) on the back	Two nights	Nocturnal movement accelerations; axial turn frequency, variability, size, duration, and velocity	Lower acceleration of nocturnal movements; similar frequency and variability but smaller size and duration of axial turns in PD as compared to HS	No significant correlations
Bhidayasiri et al., 2016 [27]	6 PD (65.5 ± 7.4) 6 HS (66.7 ± 7.8)	2.2 ± 1.1	Not performed	1 inertial device (including accelerometer and gyroscope) on the sternum	One night	Position changes; number, velocity, and acceleration of turns in bed; getting out of bed	Fewer and smaller axial rotations and position changes, slower velocity and acceleration, more episodes of getting out of bed in PD as compared to HS	Number and degree of rolling over episodes correlated with rotation velocity and acceleration
Sringean et al., 2016 [26]	19 PD (64.6 ± 7.9) 19 HS (64.3 ± 8.5)	2.5 ± 0.4	Not performed	5 inertial devices (including accelerometer and gyroscope) on the wrists, ankles, and trunk	One night	Number, velocity, acceleration, degree, and duration of turns in bed; getting out of bed; limb movements	Fewer turns in bed, smaller rotation degree, velocity, and acceleration; more episodes of getting out of bed in PD than HS; a similar amount of movement in the more and less affected arms in PD	Rotation duration, degree, velocity, and acceleration correlated with axial impairment; leg movements correlated with RBD1Q score
Bhidayasiri et al., 2016 [28]	10 PD under and not under overnight subcutaneous apomorphine infusion (65.4 ± 12.3)	3.2 ± 0.7	Not performed	1 inertial device (including accelerometer and gyroscope) on the sternum	Two nights	Number, degree, velocity, and acceleration of turns in bed; getting out of bed	Improvement in the number, velocity, and degree of turns in bed under subcutaneous apomorphine infusion	Number of turns in bed correlated with mean daily dosage of apomorphine infusion
Bhidayasiri et al., 2017 [29]	17 PD (64.9 ± 7.9) 17 HS (64.3 ± 8.6)	2.6 ± 0.4	Not performed	1 inertial device (including accelerometer and gyroscope) on the sternum	Two nights	Number, degree, velocity, and acceleration of turns in bed; torque of axial rotation in bed	Fewer episodes and reduced degree, velocity, and acceleration of turns; lower torque of axial rotations in PD than HS	The torque of turning in bed correlated with disease duration and severity
Bhidayasiri et al., 2017 [30]	17 PD treated with rotigotine (60.6 ± 9.5) 17 PD treated with placebo (63.5 ± 12.5)	2.8 ± 0.8	Not performed	1 inertial device (including accelerometer and gyroscope) on the sternum	Two nights	Number, degree, velocity and acceleration of turns in bed; getting out of bed	Rotigotine improved the number and degree of axial turns in bed	Not performed
Sringean et al., 2017 [31]	18 PD (64.9 ± 7.6) 18 HS (63.8 ± 8.5)	2.5 ± 0.4	Not performed	5 inertial devices (including accelerometer and gyroscope) on the wrists, ankles, and trunk	One night	Number, velocity, acceleration, degree, and duration of turns in bed; getting out of bed; limb movements; sleep positions in the first and second half of the night	Fewer episodes, reduced speed, acceleration, and degree of turns; a similar number of getting-out episodes and limb movements, more time spent in the supine position in PD as compared with HS; more prominent changes during the second half of the night in PD	Time spent in supine position correlated with axial motor impairment and degree of turns in bed
Uchino et al., 2017 [32]	64 PD (73.3 ± 8.2) No HS	3 ± 1	Not performed	1 triaxial accelerometer on the abdomen	One night	Total time in bed and in the supine position; number of turnover movements; mean interval between turnover movements	Longer time in bed and in the supine position and longer interval between turnover movements in PD with higher disease severity	Number of turnover movements in bed correlated with mobility and disease duration and severity
Xue et al., 2018 [33]	17 PD + IBM (68.5 ± 5.8) 12 PD − IBM (66.1 ± 8.5) No HS	2.3 ± 0.5	Yes	5 sensors (including accelerometers, gyroscopes, and magnetometers) on the abdomen, wrists, and ankles	One night	Degree, duration, velocity, and acceleration of turns in bed; getting out of bed; limb movements	Smaller degree of turns in bed and similar number of limb movements in PD + IBM and PD − IBM	Turns in bed negatively correlated with disease duration and axial impairment, and positively correlated with PSG measures (TST and SE) of turns
Bhidayasiri et al., 2019 [34]	25 PD (65.6 ± 10.9) 25 HS (55.0 ± 16.1)	2.6 ± 0.9	Not performed	5 inertial devices (accelerometer and gyroscope) on the wrists, ankles, and trunk	One night	Number, degree, velocity, and acceleration of turning in bed	NA (validation of a clinical questionnaire)	Number and degree of turns in bed correlated with NHQ scores
Mirelman et al., 2020 [35]	304 PD (68 ± 8.4) 205 HS (66.3 ± 13.3)	1 ± NA (*n* = 47 PD) 2 ± NA (*n* = 181 PD) 3 ± NA (*n* = 77 PD)	Not performed	1 triaxial accelerometer-based device on the waist	Two nights	Lying, turning, and sleep interruptions (percentage of night walking and upright time); number, velocity, time, side, and degree of turns in bed	A similar number, but longer duration and reduced size of rotations in early PD than HS; supine position more frequent in PD than HS; despite similar rest duration and turn degrees, advanced PD had fewer, slower, and longer turns and greater night upright time than early PD; lower turns more frequent in the late night than early night in advanced PD	Nocturnal bed rotations negatively correlated with cognitive function and non-motor symptoms; nocturnal movements positively correlated with LEDD and disease severity (e.g., rigidity, bradykinesia, and PIGD scores)

H&Y: Hoehn and Yahr; HS: healthy subjects; LEDD: L-Dopa equivalent daily dose; NA: not available; NHQ: Nocturnal Hypokinesia Questionnaire; OFF state: not on dopaminergic therapy; ON state: on dopaminergic therapy; PD: Parkinson’s disease patients; PD + IBM: patients with Parkinson’s disease and subjective impaired bed mobility; PD − IBM: patients with Parkinson’s disease without subjective impaired bed mobility; PIGD: postural instability/gait difficulty; PSG: polysomnography; RBD1Q: REM Sleep Behaviour Disorder Single-Question Screen; SD: standard deviation; TST: total sleep time.

## Abbreviations

BAN	Body area network
EEG	Electroencephalography
EOG	Electrooculography
HS	Healthy subjects
IMU	Inertial measurement unit
NREM	Non-rapid eye movement
PD	Parkinson’s disease
PLMD	Periodic limb movement disorder
RBD	REM behaviour disorder
REM	Rapid eye movement
RLS	Restless legs syndrome
sEMG	Surface electromyography
SNpc	Substantia nigra pars compacta

## References

[1] GBD 2016 Parkinson’s Disease Collaborators (2018). Global, regional, and national burden of Parkinson’s disease, 1990–2016: A systematic analysis for the Global Burden of Disease Study 2016. Lancet Neurol..

[2] Postuma R.B., Berg D., Stern M., Poewe W., Olanow C.W., Oertel W., Obeso J., Marek K., Litvan I., Lang A.E. (2015). MDS clinical diagnostic criteria for Parkinson’s disease. Mov. Disord..

[3] Poewe W. (2008). Non-motor symptoms in Parkinson’s disease. Eur. J. Neurol..

[4] Armstrong M.J., Okun M.S. (2020). Diagnosis and Treatment of Parkinson Disease: A Review. JAMA.

[5] Suppa A., Bologna M., Conte A., Berardelli A., Fabbrini G. (2017). The effect of L-dopa in Parkinson’s disease as revealed by neurophysiological studies of motor and sensory functions. Expert Rev. Neurother..

[6] Timpka J., Nitu B., Datieva V., Odin P., Antonini A. (2017). Device-Aided Treatment Strategies in Advanced Parkinson’s Disease. Int. Rev. Neurobiol..

[7] Bhidayasiri R., Sringean J., Trenkwalder C. (2020). Mastering nocturnal jigsaws in Parkinson’s disease: A dusk-to-dawn review of night-time symptoms. J. Neural. Transm. (Vienna).

[8] Hely M.A., Morris J.G.L., Reid W.G.J., Trafficante R. (2005). Sydney multicenter study of Parkinson’s disease: Non-L-dopa–responsive problems dominate at 15 years. Mov. Disord..

[9] Bhidayasiri R., Trenkwalder C. (2018). Getting a good night sleep? The importance of recognizing and treating nocturnal hypokinesia in Parkinson’s disease. Parkinsonism Relat. Disord..

[10] Videnovic A., Golombek D. (2013). Circadian and sleep disorders in Parkinson’s disease. Exp. Neurol..

[11] Chahine L., Amara A., Videnovic A. (2017). A Systematic Review of the Literature on Disorders of Sleep and Wakefulness in Parkinson’s Disease From 2005–2015. Sleep Med. Rev..

[12] Jean-Louis G., Kripke D.F., Mason W.J., Elliott J.A., Youngstedt S.D. (2001). Sleep estimation from wrist movement quantified by different actigraphic modalities. J. Neurosci. Methods.

[13] Barnes J., Connelly V., Wiggs L., Boubert L., Maravic K. (2010). Sleep patterns in Parkinson’s disease patients with visual hallucinations. Int. J. Neurosci..

[14] Baumann-Vogel H., Imbach L.L., Sürücü O., Stieglitz L., Waldvogel D., Baumann C.R., Werth E. (2017). The Impact of Subthalamic Deep Brain Stimulation on Sleep-Wake Behavior: A Prospective Electrophysiological Study in 50 Parkinson Patients. Sleep.

[15] Comella C.L., Morrissey M., Janko K. (2005). Nocturnal activity with nighttime pergolide in Parkinson disease: A controlled study using actigraphy. Neurology.

[16] Giganti F., Ramat S., Zilli I., Guidi S., Raglione L.M., Sorbi S., Salzarulo P. (2013). Daytime course of sleepiness in de novo Parkinson’s disease patients. J. Sleep Res..

[17] Louter M., van Sloun R.J.G., Pevernagie D.A.A., Arends J.B.A.M., Cluitmans P.J., Bloem B.R., Overeem S. (2013). Subjectively impaired bed mobility in Parkinson disease affects sleep efficiency. Sleep Med..

[18] Louter M., Arends J.B.A.M., Bloem B.R., Overeem S. (2014). Actigraphy as a diagnostic aid for REM sleep behavior disorder in Parkinson’s disease. BMC Neurol..

[19] Naismith S.L., Rogers N.L., Mackenzie J., Hickie I.B., Lewis S.J.G. (2010). The relationship between actigraphically defined sleep disturbance and REM sleep behaviour disorder in Parkinson’s Disease. Clin. Neurol. Neurosurg..

[20] Madrid-Navarro C.J., Puertas Cuesta F.J., Escamilla-Sevilla F., Campos M., Ruiz Abellán F., Rol M.A., Madrid J.A. (2019). Validation of a Device for the Ambulatory Monitoring of Sleep Patterns: A Pilot Study on Parkinson’s Disease. Front Neurol..

[21] Nass A., Nass R.D. (2008). Actigraphic evidence for night-time hyperkinesia in Parkinson’s disease. Int. J. Neurosci..

[22] Prudon B., Duncan G.W., Khoo T.K., Yarnall A.J., Anderson K.N. (2014). Primary sleep disorder prevalence in patients with newly diagnosed Parkinson’s disease. Mov. Disord..

[23] van Hilten B., Hoff J.I., Middelkoop H.A., van der Velde E.A., Kerkhof G.A., Wauquier A., Kamphuisen H.A., Roos R.A. (1994). Sleep disruption in Parkinson’s disease. Assessment by continuous activity monitoring. Arch. Neurol..

[24] Whitehead D.L., Davies A.D.M., Playfer J.R., Turnbull C.J. (2008). Circadian rest-activity rhythm is altered in Parkinson’s disease patients with hallucinations. Mov. Disord..

[25] Louter M., Maetzler W., Prinzen J., van Lummel R.C., Hobert M., Arends J.B.A.M., Bloem B.R., Streffer J., Berg D., Overeem S. (2015). Accelerometer-based quantitative analysis of axial nocturnal movements differentiates patients with Parkinson’s disease, but not high-risk individuals, from controls. J. Neurol. Neurosurg. Psychiatry.

[26] Sringean J., Taechalertpaisarn P., Thanawattano C., Bhidayasiri R. (2016). How well do Parkinson’s disease patients turn in bed? Quantitative analysis of nocturnal hypokinesia using multisite wearable inertial sensors. Park. Relat. Disord..

[27] Bhidayasiri R., Sringean J., Taechalertpaisarn P., Thanawattano C. (2016). Capturing nighttime symptoms in Parkinson disease: Technical development and experimental verification of inertial sensors for nocturnal hypokinesia. J. Rehabil. Res. Dev..

[28] Bhidayasiri R., Sringean J., Anan C., Boonpang K., Thanawattano C., Ray Chaudhuri K. (2016). Quantitative demonstration of the efficacy of night-time apomorphine infusion to treat nocturnal hypokinesia in Parkinson’s disease using wearable sensors. Park. Relat. Disord..

[29] Bhidayasiri R., Sringean J., Thanawattano C. (2017). Impaired bed mobility: Quantitative torque analysis with axial inertial sensors. Neurodegener. Dis. Manag..

[30] Bhidayasiri R., Sringean J., Chaiwong S., Anan C., Penkeaw N., Leaknok A., Boonpang K., Saksornchai K., Rattanachaisit W., Thanawattano C. (2017). Rotigotine for nocturnal hypokinesia in Parkinson’s disease: Quantitative analysis of efficacy from a randomized, placebo-controlled trial using an axial inertial sensor. Park. Relat. Disord..

[31] Sringean J., Anan C., Thanawattano C., Bhidayasiri R. (2017). Time for a strategy in night-time dopaminergic therapy? An objective sensor-based analysis of nocturnal hypokinesia and sleeping positions in Parkinson’s disease. J. Neurol. Sci..

[32] Uchino K., Shiraishi M., Tanaka K., Akamatsu M., Hasegawa Y. (2017). Impact of inability to turn in bed assessed by a wearable three-axis accelerometer on patients with Parkinson’s disease. PLoS ONE.

[33] Xue F., Wang F.Y., Mao C.J., Guo S.-P., Chen J., Li J., Wang Q.J., Bei H.Z., Yu Q., Liu C.F. (2018). Analysis of nocturnal hypokinesia and sleep quality in Parkinson’s disease. J. Clin. Neurosci..

[34] Bhidayasiri R., Phokaewvarangkul O., Sringean J., Martinez-Martin P., Anan C., Kantachadvanich N., Chaudhuri K.R., Hattori N. (2019). Evaluation of nocturnal hypokinesia in Parkinson’s disease using a novel patient/proxy questionnaire and correlations with objective monitoring. Park. Relat. Disord..

[35] Mirelman A., Hillel I., Rochester L., Del Din S., Bloem B.R., Avanzino L., Nieuwboer A., Maidan I., Herman T., Thaler A. (2020). Tossing and Turning in Bed: Nocturnal Movements in Parkinson’s Disease. Mov. Disord..

[36] Bhidayasiri R., Sringean J., Thanawattano C. (2016). Sensor-based evaluation and treatment of nocturnal hypokinesia in Parkinson’s disease: An evidence-based review. Park. Relat. Disord..

[37] Wilde-Frenz J., Schulz H. (1983). Rate and distribution of body movements during sleep in humans. Percept. Mot. Skills.

[38] Patel A.K., Reddy V., Araujo J.F. (2020). Physiology, Sleep Stages. StatPearls.

[39] Chase M.H. (2013). Motor control during sleep and wakefulness: Clarifying controversies and resolving paradoxes. Sleep Med. Rev..

[40] De Koninck J., Gagnon P., Lallier S. (1983). Sleep positions in the young adult and their relationship with the subjective quality of sleep. Sleep.

[41] Hobson J.A., Spagna T., Malenka R. (1978). Ethology of sleep studied with time-lapse photography: Postural immobility and sleep-cycle phase in humans. Science.

[42] Trotti L.M. (2017). Restless Legs Syndrome and Sleep-Related Movement Disorders. Continuum.

[43] Stefani A., Gabelia D., Mitterling T., Poewe W., Högl B., Frauscher B. (2015). A Prospective Video-Polysomnographic Analysis of Movements during Physiological Sleep in 100 Healthy Sleepers. Sleep.

[44] Manni R., Terzaghi M. (2005). Rhythmic movements during sleep: A physiological and pathological profile. Neurol. Sci..

[45] De Koninck J., Lorrain D., Gagnon P. (1992). Sleep positions and position shifts in five age groups: An ontogenetic picture. Sleep.

[46] Skarpsno E.S., Mork P.J., Nilsen T.I.L., Holtermann A. (2017). Sleep positions and nocturnal body movements based on free-living accelerometer recordings: Association with demographics, lifestyle, and insomnia symptoms. Nat. Sci. Sleep.

[47] Gori S., Ficca G., Giganti F., Di Nasso I., Murri L., Salzarulo P. (2004). Body movements during night sleep in healthy elderly subjects and their relationships with sleep stages. Brain Res. Bull..

[48] Stefani A., Högl B. (2020). Sleep in Parkinson’s disease. Neuropsychopharmacology.

[49] Dauer W., Przedborski S. (2003). Parkinson’s disease: Mechanisms and models. Neuron.

[50] Lang A.E., Lozano A.M. (1998). Parkinson’s disease. First of two parts. N. Engl. J. Med..

[51] Anderson E., Nutt J. (2011). The long-duration response to levodopa: Phenomenology, potential mechanisms and clinical implications. Park. Relat. Disord..

[52] Dauvilliers Y., Schenck C.H., Postuma R.B., Iranzo A., Luppi P.-H., Plazzi G., Montplaisir J., Boeve B. (2018). REM sleep behaviour disorder. Nat. Rev. Dis. Prim..

[53] Nagandla K., De S. (2013). Restless legs syndrome: Pathophysiology and modern management. Postgrad. Med. J..

[54] Trenkwalder C., Walters A.S., Hening W. (1996). Periodic limb movements and restless legs syndrome. Neurol. Clin..

[55] Braak H., Del Tredici K., Rüb U., de Vos R.A.I., Jansen Steur E.N.H., Braak E. (2003). Staging of brain pathology related to sporadic Parkinson’s disease. Neurobiol. Aging.

[56] Olson E.J., Boeve B.F., Silber M.H. (2000). Rapid eye movement sleep behaviour disorder: Demographic, clinical and laboratory findings in 93 cases. Brain.

[57] Alonso-Navarro H., García-Martín E., Agúndez J.A.G., Jiménez-Jiménez F.J. (2019). Association between restless legs syndrome and other movement disorders. Neurology.

[58] Zucconi M., Ferri R., Allen R., Baier P.C., Bruni O., Chokroverty S., Ferini-Strambi L., Fulda S., Garcia-Borreguero D., Hening W.A. (2006). The official World Association of Sleep Medicine (WASM) standards for recording and scoring periodic leg movements in sleep (PLMS) and wakefulness (PLMW) developed in collaboration with a task force from the International Restless Legs Syndrome Study Group (IRLSSG). Sleep Med..

[59] Kurtis M.M., Balestrino R., Rodriguez-Blazquez C., Forjaz M.J., Martinez-Martin P. (2018). A Review of Scales to Evaluate Sleep Disturbances in Movement Disorders. Front. Neurol..

[60] Rundo J.V., Downey R. (2019). Polysomnography. Handb. Clin. Neurol..

[61] Hirshkowitz M. (2016). Polysomnography Challenges. Sleep Med. Clin..

[62] Fekedulegn D., Andrew M.E., Shi M., Violanti J.M., Knox S., Innes K.E. (2020). Actigraphy-Based Assessment of Sleep Parameters. Ann. Work Expo. Health.

[63] Ancoli-Israel S., Cole R., Alessi C., Chambers M., Moorcroft W., Pollak C.P. (2003). The role of actigraphy in the study of sleep and circadian rhythms. Sleep.

[64] Chen M., Gonzalez S., Vasilakos A., Cao H., Leung V.C. (2011). Body Area Networks: A Survey. Mob. Netw. Appl..

[65] Jovanov E., Hanish N., Courson V., Stidham J., Stinson H., Webb C., Denny K. Avatar—A multi-sensory system for real time body position monitoring. Proceedings of the 2009 Annual International Conference of the IEEE Engineering in Medicine and Biology Society.

[66] Mazzetta I., Gentile P., Pessione M., Suppa A., Zampogna A., Bianchini E., Irrera F. (2018). Stand-Alone Wearable System for Ubiquitous Real-Time Monitoring of Muscle Activation Potentials. Sensors (Basel).

[67] Mazzetta I., Zampogna A., Suppa A., Gumiero A., Pessione M., Irrera F. (2019). Wearable Sensors System for an Improved Analysis of Freezing of Gait in Parkinson’s Disease Using Electromyography and Inertial Signals. Sensors (Basel).

[68] Zampogna A., Mileti I., Palermo E., Celletti C., Paoloni M., Manoni A., Mazzetta I., Dalla Costa G., Pérez-López C., Camerota F. (2020). Fifteen Years of Wireless Sensors for Balance Assessment in Neurological Disorders. Sensors (Basel).

[69] Stavitsky K., Saurman J.L., McNamara P., Cronin-Golomb A. (2010). Sleep in Parkinson’s disease: A comparison of actigraphy and subjective measures. Park. Relat. Disord..

[70] Niwa F., Kuriyama N., Nakagawa M., Imanishi J. (2011). Circadian rhythm of rest activity and autonomic nervous system activity at different stages in Parkinson’s disease. Auton. Neurosci..

[71] Stavitsky K., Neargarder S., Bogdanova Y., McNamara P., Cronin-Golomb A. (2012). The impact of sleep quality on cognitive functioning in Parkinson’s disease. J. Int. Neuropsychol. Soc..

[72] Wienecke M., Werth E., Poryazova R., Baumann-Vogel H., Bassetti C.L., Weller M., Waldvogel D., Storch A., Baumann C.R. (2012). Progressive dopamine and hypocretin deficiencies in Parkinson’s disease: Is there an impact on sleep and wakefulness?. J. Sleep Res..

[73] Maglione J.E., Liu L., Neikrug A.B., Poon T., Natarajan L., Calderon J., Avanzino J.A., Corey-Bloom J., Palmer B.W., Loredo J.S. (2013). Actigraphy for the Assessment of Sleep Measures in Parkinson’s Disease. Sleep.

[74] Aitken D., Naismith S.L., Terpening Z., Lewis S.J.G. (2014). Dysfunctional sleep beliefs in Parkinson’s disease: Relationships with subjective and objective sleep. J. Clin. Neurosci..

[75] Bolitho S.J., Naismith S.L., Rajaratnam S.M.W., Grunstein R.R., Hodges J.R., Terpening Z., Rogers N., Lewis S.J.G. (2014). Disturbances in melatonin secretion and circadian sleep-wake regulation in Parkinson disease. Sleep Med..

[76] Breen D.P., Vuono R., Nawarathna U., Fisher K., Shneerson J.M., Reddy A.B., Barker R.A. (2014). Sleep and circadian rhythm regulation in early Parkinson disease. JAMA Neurol..

[77] Gunn D.G., Naismith S.L., Terpening Z., Lewis S.J.G. (2014). The Relationships Between Poor Sleep Efficiency and Mild Cognitive Impairment in Parkinson Disease. J. Geriatr. Psychiatry Neurol..

[78] Gunn D.G., Naismith S.L., Bolitho S.J., Lewis S.J.G. (2014). Actigraphically-defined sleep disturbance in Parkinson’s disease is associated with differential aspects of cognitive functioning. J. Clin. Neurosci..

[79] Kotschet K., Johnson W., McGregor S., Kettlewell J., Kyoong A., O’Driscoll D.M., Turton A.R., Griffiths R.I., Horne M.K. (2014). Daytime sleep in Parkinson’s disease measured by episodes of immobility. Park. Relat. Disord..

[80] Klingelhoefer L., Rizos A., Sauerbier A., McGregor S., Martinez-Martin P., Reichmann H., Horne M., Chaudhuri K.R. (2016). Night-time sleep in Parkinson’s disease-the potential use of Parkinson’s KinetiGraph: A prospective comparative study. Eur. J. Neurol..

[81] Bargiotas P., Eugster L., Oberholzer M., Debove I., Lachenmayer M.L., Mathis J., Pollo C., Schüpbach W.M.M., Bassetti C.L. (2017). Sleep-wake functions and quality of life in patients with subthalamic deep brain stimulation for Parkinson’s disease. PLoS ONE.

[82] Prasad V., Brown C.A. (2017). A Pilot Study to Determine the Consistency of Simultaneous Sleep Actigraphy Measurements Comparing All Four Limbs of Patients with Parkinson Disease. Geriatrics (Basel).

[83] Madrid-Navarro C.J., Escamilla-Sevilla F., Mínguez-Castellanos A., Campos M., Ruiz-Abellán F., Madrid J.A., Rol M.A. (2018). Multidimensional Circadian Monitoring by Wearable Biosensors in Parkinson’s Disease. Front. Neurol..

[84] Kataoka H., Saeki K., Kurumatani N., Sugie K., Obayashi K. (2020). Objective sleep measures between patients with Parkinson’s disease and community-based older adults. Sleep Med..

[85] Wu J.Q., Cronin-Golomb A. (2020). Temporal Associations between Sleep and Daytime Functioning in Parkinson’s Disease: A Smartphone-Based Ecological Momentary Assessment. Behav. Sleep Med..

[86] Kataoka H., Saeki K., Yamagami Y., Sugie K., Obayashi K. (2020). Quantitative associations between objective sleep measures and early-morning mobility in Parkinson’s disease: Cross-sectional analysis of the PHASE study. Sleep.

[87] Stavitsky K., Cronin-Golomb A. (2011). Sleep quality in Parkinson disease: An examination of clinical variables. Cogn. Behav. Neurol..

[88] Bolitho S.J., Naismith S.L., Salahuddin P., Terpening Z., Grunstein R.R., Lewis S.J.G. (2013). Objective measurement of daytime napping, cognitive dysfunction and subjective sleepiness in Parkinson’s disease. PLoS ONE.

[89] Dowling G.A., Mastick J., Colling E., Carter J.H., Singer C.M., Aminoff M.J. (2005). Melatonin for sleep disturbances in Parkinson’s disease. Sleep Med..

[90] van Dijk K.D., Møst E.I.S., Van Someren E.J.W., Berendse H.W., van der Werf Y.D. (2009). Beneficial effect of transcranial magnetic stimulation on sleep in Parkinson’s disease. Mov. Disord..

[91] Arias P., Vivas J., Grieve K.L., Cudeiro J. (2010). Double-blind, randomized, placebo controlled trial on the effect of 10 days low-frequency rTMS over the vertex on sleep in Parkinson’s disease. Sleep Med..

[92] Perez-Lloret S., Santiago P.-L., Rossi M., Cardinali D.P., Merello M. (2010). Activity-rest rhythm abnormalities in Parkinson’s disease patients are related to dopaminergic therapy. Int. J. Neurosci..

[93] Rios Romenets S., Creti L., Fichten C., Bailes S., Libman E., Pelletier A., Postuma R.B. (2013). Doxepin and cognitive behavioural therapy for insomnia in patients with Parkinson’s disease–A randomized study. Park. Relat. Disord..

[94] Videnovic A., Klerman E.B., Wang W., Marconi A., Kuhta T., Zee P.C. (2017). Timed Light Therapy for Sleep and Daytime Sleepiness Associated With Parkinson Disease: A Randomized Clinical Trial. JAMA Neurol..

[95] Perez Lloret S., Rossi M., Cardinali D.P., Merello M. (2008). Validation of the sleep related items of the Non-motor Symptoms Questionnaire for Parkinson’s disease (NMSQuest). Park. Relat. Disord..

[96] Perez-Lloret S., Rossi M., Nouzeilles M.I., Trenkwalder C., Cardinali D.P., Merello M. (2009). Parkinson’s disease sleep scale, sleep logs, and actigraphy in the evaluation of sleep in parkinsonian patients. J. Neurol..

[97] Yoneyama M., Mitoma H., Okuma Y. (2013). Accelerometry-based long-term monitoring of movement disorders: From diurnal gait behavior to nocturnal bed mobility. J. Mech. Med. Biol..

[98] Yoneyama M., Kurihara Y., Watanabe K., Mitoma H. (2013). Accelerometry-based gait analysis and its application to Parkinson’s disease assessment-part 2: A new measure for quantifying walking behavior. IEEE Trans. Neural. Syst. Rehabil. Eng..

[99] Cochen De Cock V., Benard-Serre N., Driss V., Granier M., Charif M., Carlander B., Desplan M., Croisier Langenier M., Cugy D., Bayard S. (2015). Supine sleep and obstructive sleep apnea syndrome in Parkinson’s disease. Sleep Med..

[100] Boe A.J., McGee Koch L.L., O’Brien M.K., Shawen N., Rogers J.A., Lieber R.L., Reid K.J., Zee P.C., Jayaraman A. (2019). Automating sleep stage classification using wireless, wearable sensors. NPJ Digit. Med..

[101] Casson A.J. (2019). Wearable EEG and beyond. Biomed. Eng. Lett..

[102] Chow H.W., Yang C.C. (2020). Accuracy of Optical Heart Rate Sensing Technology in Wearable Fitness Trackers for Young and Older Adults: Validation and Comparison Study. JMIR Mhealth Uhealth.

[103] Boudreau P., Yeh W.H., Dumont G.A., Boivin D.B. (2013). Circadian Variation of Heart Rate Variability across Sleep Stages. Sleep.

[104] Goud K.Y., Moonla C., Mishra R.K., Yu C., Narayan R., Litvan I., Wang J. (2019). Wearable Electrochemical Microneedle Sensor for Continuous Monitoring of Levodopa: Toward Parkinson Management. ACS Sens..

